# Assessing Walking Ability in People with HTLV-1-Associated Myelopathy Using the 10 Meter Timed Walk and the 6 Minute Walk Test

**DOI:** 10.1371/journal.pone.0157132

**Published:** 2016-06-23

**Authors:** Adine Adonis, Graham P. Taylor

**Affiliations:** 1 National Centre for Human Retrovirology, St Mary’s Hospital, Imperial College Healthcare NHS Trust, London, W2 1NY, United Kingdom; 2 Section of Virology, Department of Medicine, Imperial College, London, W2 1PG, United Kingdom; Rutgers University -New Jersey Medical School, UNITED STATES

## Abstract

**Background:**

Five to ten million persons, are infected by HTLV-1 of which 3% will develop HTLV-1-associated myelopathy (HAM) a chronic, disabling inflammation of the spinal cord. Walking, a fundamental, complex, multi-functional task is demanding of multiple body systems. Restricted walking ability compromises activity and participation levels in people with HAM (pwHAM). Therapy aims to improve mobility but validated measures are required to assess change.

**Study Design:**

Prospective observational study.

**Objectives:**

To explore walking capacity in pwHAM, walking endurance using the 6 minute walk (6MW), and gait speed, using the timed 10m walk (10mTW).

**Setting:**

Out-patient setting in an inner London Teaching hospital.

**Methods:**

Prospective documentation of 10mTW and 6MW distance; walking aid usage and pain scores measured twice, a median of 18 months apart.

**Results:**

Data analysis was completed for twenty-six pwHAM, (8♂; 18♀; median age: 57.8 years; median disease duration: 8 years). Median time at baseline to: complete 10m was 17.5 seconds, versus 21.4 seconds at follow up; 23% completed the 6MW compared to 42% at follow up and a median distance of 55m was covered compared to 71m at follow up. Using the 10mTW velocity to predict the 6MW distance, overestimated the distance walked in 6 minutes (p<0.01). Functional decline over time was captured using the functional ambulation categories.

**Conclusions:**

The 10mTW velocity underestimated the degree of disability. Gait speed usefully predicts functional domains, shows direction of functional change and comparison with published healthy age matched controls show that these patients have significantly slower gait speeds. The measured differences over 18 months were sufficient to reliably detect change and therefore these assessments can be useful to detect improvement or deterioration within broader disability grades. Walking capacity in pwHAM should be measured using the 10mTW for gait speed and the 6MW for endurance.

## Introduction

HTLV-1-associated myelopathy (HAM) is a non-remitting, slowly progressive inflammation of the spinal cord, affecting approximately 3% of the 5–10 million HTLV-1 infected individuals worldwide[1;2].The condition is characterised by proximal, more than distal, motor weakness; lower limb spasticity, pain and bladder, bowel and sexual dysfunction[3]. Activity limitations in people with HAM (pwHAM) include: walking; stair climbing; washing and dressing and bladder management[4]. The aim of disease modifying treatment is to improve or preserve function or to slow functional decline in all areas, especially walking.

HAM related disability has been assessed by several categorical scales. Aberlado and colleagues used the Functional Independence Measure /Functional Assessment Measure (FIM/FAM) to describe the disability profile of pwHAM, highlighting locomotion, stair climbing and bowel and bladder management^4^. Franzoi and Aberlado employing the Ambulation Function classification and the American Spinal Injury Association Lower Extremity Motor Scores (ASIA LEMS) identified knee extension and plantar flexion as strong correlates for community ambulation[5]. Other measures used include the Expanded Disability Status Scale (EDSS), Osame’s Disability Scale (ODS)[6] and the Evandro Chagas Clinical Research Institute (IPEC) scale[7].

Walking, a complex task, is a fundamental function facilitating independence and allowing multiple interactive experiences with the environment[8]. As a most basic activity of daily living (ADL) it places demands on the nervous, cardiovascular and musculoskeletal systems, which may singularly or collectively, have an effect on walking capacity[8;9;10]. Persons with a neurological impairment aim to remain as independent as possible and impairment of gait impacts both the level of activity and level of participation[11]. For many people with neurological conditions, walking is what they most treasure, and want to regain, improve or preserve.

Categorizing the disability profile of patients, whilst useful given the non-remitting nature of the disease, may not fully capture change to a patient’s functional independence. Further details are needed to expand our understanding of the functional impact for pwHAM.

Walking speed over a short fixed distance mainly evaluates lower limb function and is considered representative of gait quality and motor function[12;13]. Wade and colleagues were the first to describe and document the specific use of a timed 10m walking test to monitor the walking recovery of patients post stroke[14]. It is a pragmatic quick test, easily applied in most clinical settings, requires minimal equipment and has good sensitivity to changes in gait speed. Using the 10m timed walk (10mTW), Martin et al demonstrated that walking function deteriorated by an average of 4 seconds/10m/year in pwHAM[15].

The 6 minute walk test (6MWT), a modification of the 12 minute walk, was originally developed for cardiorespiratory patients to specifically evaluate and monitor their functional capacity, establish prognosis and evaluate change in disease in relation to treatment[16;17;18]. It measures walking endurance, is used as a standard assessment for functional capacity across a variety of neurological conditions. Distance walked in meters in up to 6 minutes and the time taken are recorded. 6MWT performance in pwHAM has not been documented.

Quantification of the endurance capabilities for pwHAM and understanding whether and how this complements the documented use of the 10mTW within this patient cohort, will potentially provide a means of quantifying the current functional ability of a pwHAM as it relates to their activity. By detecting early functional decline clinicians can potentially offer disease modifying treatment sooner and preserve function for longer. The objective of this study was to improve the management of pwHAM by better measurement of disease impact. The primary aim was to explore the relative contributions of the 10mTW and the 6MWT in the assessment of mobility in pwHAM and compare these with previously published healthy age matched controls and to compare the 10m TW with functional ambulation categories and functional categories.

The second aim was to determine whether these measures could detect change over the minimum time period specified.

## Methods

The National Centre for Human Retrovirology (NCHR) at St Mary’s Hospital, Imperial College Healthcare NHS Trust is the main referral centre for patients with HTLV infection in the United Kingdom (UK). Founded in 1993 the need for comprehensive documentation of disease progression to include description of pwHAM's walking pattern and use of walking aids was soon apparent. Objective measures such as the 10mTW and 6MWT were introduced to document change in gait disability and are performed at each assessment.

### Inclusion criteria

Patients were included in the study if they had HAM diagnosed according to WHO criteria(WHO 1998); had measures performed at least 11 months apart and were able to walk a minimum of 10m on both occasions.

### Exclusion Criteria

Patients with HAM were excluded if they were unable to mobilise 10m at baseline and or follow up using their usual walking aid.

The results of prospectively recorded routine clinical assessment of gait (6MTW; 10mWT and walking aids) and pain were abstracted from the case notes of pwHAM, attending the NCHR between 2009–2014. Pain was measured using a self-reporting, 11point, 10cm visual analogue scale (VAS). Potential disease modifying therapies were documented. Disease modifying therapies are defined as treatments that affect the underlying pathophysiology of the disease. For our patient cohort this would include the immunosuppressive agents: ciclosporin, methotrexate or methyl prednisolone.

The 6MWT assessment was performed at a single site using 10m repeats with turns along a 13.5 meter long, smooth, flat, uncluttered corridor and no encouragement was provided[19] with patients being instructed to: ‘walk for as long as you are able’. Patients were not provided with a rest break and once they stopped walking, irrespective of whether they completed the 6 minutes, the test was ended. In the 10mTW, the time taken (seconds) to walk 10m, with a dynamic start, in the same environment was recorded. For both assessments, gait speed was at the patient’s normal pace using their usual walking aid, no physical assistance was provided and time was recorded using a digital stopwatch. Using age and height, predicted performance for the 6MWT in the absence of disease, was calculated using reference formula for an unrepeated 6 minute walk[20]. Comparisons between the gait speed of patients with HAM were made to those of healthy age matched control data[21].

Functional ambulation categories were calculated based on the modified system proposed by Perry et al[22] for those with post stroke walking disability both at home and within the community. The functional ambulation categories, use a specific gait speed range to categorise participants from household ambulation(0–0.4m/second), the most dependent category, to limited community ambulation(>0.4m/second-0.8m/second) and finally to community ambulation(>0.8m/second-1.2m/second), which is the most independent category [22]. The functional categories related to gait speed were 0–0.2m/second indicating the need for discharge to a nursing home if hospitalized; if gait speed was greater than 0.2m/sec up to1.4m/sec then the patient could be considered for discharge home; those with gait speeds of 0–0.6 m/sec were at greater risk of falls and were dependent for their activities of daily living and those with gait speeds of 1.2m/sec-1.4m/second had normal walking speed to cross a road[23;24].There are many variables associated with safely crossing a road including, but not limited to: managing obstacles, increasing gait speed; walking in a crowd and negotiating curbs[24].

### Statistical Analysis

Anonymised data were analysed in Excel and using SPSS-20 (Chicago, Illinois). Assessment of the data for normality showed a positively skewed distribution. Descriptive statistics, intra-patient comparison of distance, time, velocity and pain at the two time points was performed using non-parametric tests (Wilcoxon’s Test), Spearmans correlation coefficients and one way ANOVA to determine inter-variable relationships and linear regression to determine interdependence of variables between the dependent variable (6MWT distance) and independent variables (10m velocity; pain; walking aid; anti-inflammatory treatment) were performed. A p value of 0.05 or less was considered statistically significant.

### Ethics

In the United Kingdom clinical research is governed by the National Research Ethics Service. The current study was conducted under the auspices of Communicable Disease Group Research Tissue Bank which was approved by NRES reference 09/h0606/106. In this research project patients consent to the collection of clinical samples, and the utilisation of their clinical data for research. Patients attending the National Centre for Human Retrovirology are provided with an information sheet about this research and all participants included in the study provided written informed consent.

## Results

During 2009–2014 26 pwHAM met the assessment criteria. Median age was 57.8 years (**Range**: 26–83.3 years); 51.2% were Afro-Caribbean, 23.8% White and 21.4% other ethnicity. Median disease duration was 8 years (**Range**: 1–27 years). The median interval between the analysed tests was 18 months.

### Baseline 10mTW

The median 10mTW time was 17.45seconds (Table 1). The median velocity of 0.6 meters/second (m/s) was significantly slower compared to 1.4 m/s of published healthy age matched controls (p = <0.001) (Table 1). Velocity varied according to walking aid usage:1.0m/s for those walking unaided; 0.5m/s for patients using one walking stick and 0.4 m/s for those using two walking sticks or a walking frame. In this cohort of pwHAM, over a distance of 10 meters: 9/26 (35%) had a median velocity of 0.3m/s classifying them as household ambulators; 9/26 (35%) had a median velocity of 0.6m/s—limited community ambulators and 8/26 (31%) had a median velocity of 1.0m/sec—community ambulators (Table 2). With walking speeds of 0–0.6m/s 53% were deemed to have a falls risk, being 1.5 times more likely to fall compared to healthy community dwelling people, including being dependent for ADLs. Only six pwHAM met or exceeded 1.0m/s which is the recognised cut off velocity for healthy individuals. If hospitalised, 92% of this cohort, by virtue of having walking speeds between 0.2–1.4m/s would be discharged home whilst the 8% with 0–0.2m/s walking speeds would be discharged to a nursing home. Only three, with walking speeds of 1.2–1.4m/s were sufficient agile to safely cross a street (Table 3).

**Table 1:** Descriptive Data Analysis of all 26 individuals.

Variable	1^st^ Time PointRangeMedianStd Deviation (SD) Standard Error of the Mean (SEM) 95% ConfidenceInterval	2^nd^ Time Point RangeMedianStd Deviation (SD) Standard Error of the Mean (SEM) 95% Confidence Interval	% ChangeRangeStd Deviation (SD) Standard Error of the Mean (SEM)	P Value
**6 Minute Walkdistance covered in meters**	10m-391m**Median**:55m**SEM**:19.61**95% CI**: 55.77–136.54	15-443m**Median**: 71m**SEM**: 24.50**95% CI**:78.05–178.99	-77.97–460%**Median**: 16m**SEM**: 29.66	**.02**
**Walking time completed in the 6min walk test in seconds**	32.20-360sec**Median**:116.80 sec**SEM**:22.54**95% CI**:127.53–220.37	37.70-360sec**Median**: 284 sec**SEM**: 21.08**95% CI**: 213.58–300.43	-63.89–757.14%**Median**: 55.3 **SEM**: 34.30	**< .01**
**Time taken to complete 10m TW**	6.70–87.80sec**Median**:17.45**SEM**:3.46**95% CI**:15.38–29.61	7.40–126.20sec**Median**:21.40**SEM**:6.46**95% CI**:18.49–45.12	-30.9–318.6%**Median**: 20.3%**SEM**:16.90	**.05**
**6 min walk Velocitym/sec**	0.10m/sec-1.10m/sec**Median**:0.50m/sec**SEM**: 0.05**95% CI**: 0.40–0.61	0.10–1.20m/sec**Median**:0.40m/sec**SEM**:0.06**95% CI**: 0.33–0.57	-79.91–39.71%**Median**: -14.10%**SEM**: 6.12	**.03**
**10m Timed Walk Velocitym/sec**	0.10m/sec-1.50m/sec**Median**:0.60m/sec**SEM**:0.07m/sec**95% CI**: 0.51–0.81	0.10m/sec-1.40m/sec**Median**:0.45m/sec**SEM**:0.07**95% CI** 0.42–0.71	-76.11–44.78%**Median**:-16.9%**SEM**: 6.07	**.04**
**Pain Categories**	0-9**Median**: 3.25**SEM**:0.74**95% CI**: 2.13–5.16	0-10**Median**: 1.50**SEM**: 0.74**95% CI**: 1.86–4.98	-100-400%**Median**: 0%**SEM**: 17.34	.74
**Disease Modifying Therapies**	1 patient ciclosporin	1 patient ciclosporin3 patients commenced methotrexate		
**Estimated 6 Minute Walk Distance Using individuals age, height, weight (Troosters et al)**	643.20m (486.40m – 880m)**Median**: 623.71**SD**:95.24**SEM**: 18.68**95% CI**: 606.63–679.86			**< .01**

**Table 2:** Functional Ambulation Categories.

Gait Speed Meters/second (m/s) /Functional Ambulation Category	Baseline N(%) Median Gait speed (m/sec)	Follow Up N(%) Median Gait speed (m/sec)	% Difference
**0–0.4m/s = Household Ambulation**	9(35%) 0.3m/s	13(50%) 0.4m/s	15%
**0.4–0.8m/s = Limited Community Ambulation**	9(35%) 0.6m/s	8(31%) 0.6m/s	-4%
**0.8–1.2m/s = Community Ambulation**	8(30%) 1.0m/s	5(19%) 1.2m/s	-11%

**Table 3:** Functional Categories.

Gait Speed Meters/second (m/s)	Functional Category	Baseline N (%)	Follow Up N (%)	% Difference
**0–0.6m/s**	Falls Risk; Dependent for Activities of Daily Living	14 (54%)	19 (73%)	19%
**0–0.2m/s**	Discharge to Nursing Home	2 (8%)	4 (15%)	7%
**0.2–1.4m/s**	Discharge Home	24 (92%)	22 (85%)	-7%
**1.2–1.4m/s**	Normal walking speed to cross a street	3 (12%)	3 (12%)	0%

### Change in 10mTW after median 18 months Follow up

The median 10mTW duration increased to 21.4 seconds, a deterioration of 4 seconds (p 0.05) with the median 10mTW velocity decreasing by 25% to 0.45m/s (p 0.04). The walking speed for those walking unaided (1.0m/s) and for those using one walking stick (0.5m/s) remain unchanged; for those using two walking sticks velocity slowed from 0.4 to 0.3m/s and for those using walking frames to 0.2m/s. Five cohort members met or exceeded the recognised healthy cut-off speed of 1.0m/s. The number of patients classified as household ambulators increased to 13, with an increase of 0.1m/sec in the median velocity. One less patient was classified as a limited community ambulator, those classified as community ambulators decreased to five but had an increased median walking speed of 1.2m/sec (Table 2). The number of patients deemed at falls risk increased by 20%. If hospitalized 8% fewer pwHAM would be discharged home (Table 3). Still only three patients had sufficient walking speed to safely cross a street (Table 3).

### 6 minute walk at Baseline (Table 1)

The median distance of 55m with a median walking time of 116.8s resulted in a median velocity of 0.5 m/s. Using age, height and weight the expected median distance walked should be 623.7m whereas the 6MWT distance based on the 10mTW velocity was predicted to be 260.5m (p = <0.01). Thus, compared with predicted values the functional exercise capacity of the cohort, as measured by the 6MWT, is severely impaired (p = <0.01). 23% of the cohort completed the 6MWT.

### 6 minute walk at Follow up (Table 1)

The median distance walked had significantly increased by 16m to 71m (p 0.02). The median time spent walking increased by 167.2s (p = <0.001) to 284s. The median predicted distance in the 6MWT, based on 10mTW velocity was 169.6m. An additional 19% of the cohort, 42% in total, completed 6 minutes however velocity had decreased by 0.1m/s to 0.4 m/s. Calculating the predicted 6MW distance using the patient’s 10mTW velocity, overestimates the actual distance patients walked in 6 minutes at both time points (p = <0.001; p = <0.001). With an intra-class correlation coefficient of 0.83, the 6MW shows good reliability over time.

The median increase in the 6 minute walk distance between the 2 time points could have been skewed by the 6 pwHAM who had significantly increased their walking distance over the 2 time points. Without these 6 patients, the median 6 minute walk distance between the 2 time points decreased by a median of 10.8m.

At both time points the 10mTW strongly, significantly, inversely, correlated with the 6MWT distance covered (r^s^ = -0.78 p = <0.001;r^s^ = -0.71 p = <0.001) and the 6MW velocity (r^s^ = -0.95 p = <0.001; r^s^ = -0.92 p = <0.001), indicating that a deteriorating 10mTW resulted in less distance covered for the 6 minute walk (Table 4). [Results reported separately for baseline and 18 month assessments].

**Table 4:** Correlation Analysis at the 2 time points (N = 26).

Spearman’s Rho	Time Point(T)	6 Min Walk Distance	Pain	Timed 10m Velocity	6 Min Walk Velocity	Disease Modifying Treatment
**Timed 10m Walk**	T1	r = -.78p = <0.01*	r = -.20p = .31		r = -.95p = <0.01*	r = .30p = .12
	T2	r = -.71p = <0.01*	r = -.05p = .79		r = -.92p = <0.01*	r = .20p = .32
**6 Min Walk Distance**	T1		r = .10p = .61	r = .75p = <0.01*		r = -.38p = .04*
	T2		r = -.12p = .53	r = .71p = <0.01*		r = -.07p = .71
**Pain**	T1			r = .21p = .28	r = .08p = .67	r = -.03p = .88
	T2			r = -.02p = .89	r = .03p = .86	r = .22p = .26
**Timed 10m Velocity**	T1				r = .93p = <0.01*	r = -.32p = .10
	T2				r = .94p = <0.01*	r = -.23p = .25

*. Correlation is significant at the 0.05 level (2-tailed) r < = 0.3: Weak correlation; r> = 0.5: Moderate Correlation; r > = 0.7: Strong Correlation

The velocities of both the timed 10mTW and the 6MWT, significantly and strongly correlated at both time points (r^s^ = .93 p = <0.001; r^s^ = .77 p = <0.001)(Table 4).The stepwise forward regression prediction model for the 6MW distance was statistically significant, accounting at baseline for 32% (F = 0.13; p = <0.001) of the variance and 63% (F = 0.41; p = <0.001) by follow up.

There were weak, non-significant correlations, for age (r^s^ = .00 p = .95;r^s^ = .01 p = .95); height (r^s^ = .10 p = .62;r^s^ = .00 p = .98); weight (r^s^ = -.04 p = .84; r^s^ = -.22;p = .28) and duration of disease (r^s^ = -.29 p = .14; r^s^ = -.36 p = .07) with the 6MW distance(Table 4). The one way ANOVA for gender and the distance walked in the 6 minute walk test was not significant at either time point (p = 0.78; F0.74; p = 0.72;F = 0.12).

Between baseline and follow up:19 pwHAM continued using the same walking aid; 2 pwHAM needed less help walking (i.e. from using 2 crutches to a walking stick) and 5 pwHAM needed more help to walk progressing to a more dependent walking aid category.

Walking aids strongly and inversely correlated with the 6MW velocity (r^s^ = -.78 p = <0.001; r^s^ = -.86 p = <0.001) and 10mTW velocity (r^s^ = -.77 p = <0.001; r^s^ = -.87 p = <0.001). (Table 4).

The use of a walking aid impacted on the 6MW distance covered at time point 1(p = 0.04) and more so at time point 2; p = 0.009). By the second time point 7 patients had changed their walking aid, 2 of the 7 patients needed less help to walk and 5 of the 7 patients needed more help to walk.

### Disease Modifying treatment (DMT)

Only 4 patients were taking disease modifying therapy at either assessment point 1 patient on ciclosporin throughout; 3 patients had initiated methotrexate by the second time point. These numbers are too small for separate analysis.

### Pain

Pain was not a significant variable throughout.

## Discussion

The data demonstrate the value that gait speed and its stratification into functional categories using velocity cut off points can add to the assessment of the walking ability of pwHAM. The 6MWT captures the poor functional walking capacity in this cohort of pwHAM. Linking walking speed and walking distance to function allows interpretation against a meaningful reference framework for comparison of functional status across patient populations and the ability to monitor function[19;23;24;25].

### Gait Speed

The data are consistent with the Martin et al[15] study demonstrating a median 4 second deterioration of the 10mTW over 18 months. This deterioration was meaningfully illustrated when patients were stratified into functional ambulation categories. Over time, despite a seemingly gradual deterioration in the 10mTW, the functional impact is significant, highlighted by the direction of transition and therefore deterioration, from community ambulation to the dependent household ambulation category. Despite having the highest level of functional ambulation, the median gait speed only reached 1.0m/s, which is the cut-off for healthy individuals. When using ambulation categories to compare across condition types, the gait speed in this cohort was slower (0.3m/s) than Kempen and colleagues’ patients with MS (0.5m/s) for those in the household ambulation category despite both groups evenly matched (pwHAM 1.0–1.2m/s and pwMS 1.3m/s) within the community ambulators[11].

Functional impairment is inversely associated with the odds of disability in both personal and domestic ADL’s[8;9;26,27;28;29;30;31;32,33,34].For those individuals who are already functionally impaired, 0.6m/s is a cut off for identifying risk of further functional decline[26;28]. Applying this cut off to our pwHAM emphasizes their functional dependence and the need for assistance with ADL’s for the majority of the cohort. The 0.2m/s median deterioration of gait speed in our pwHAM, is double the proposed minimal detectable change value of 0.1m/s for those who lack normal gait speed; and similar to pwMS[8;35].

Patient’s functional ability can further be explored by using gait speed (<0.7m/s) to predict falls risk and up to 70% of our pwHAM, had a 1.5 times increased risk of falls[24]. This risk is higher than the published prevalence of falls in mobile pwHAM (53%) and in older adults (54%)[29;36;37].

Only three patients, at either time point, had sufficient velocity (1.2–1.4m/s), to cross a road safely[23]. This velocity does not necessarily take into account the complex dynamics of stepping off a curb, walking in a crowd, paying attention to obstacles and traffic which are components of dual tasking and may further reduce walking speed[24].

A systematic review assessing walking speed in clinical research, revealed that despite its common use as an outcome measure it is limited by variance in how it is conducted[13]. The results show the advantage of using the 10mTW velocity to highlight functional decline, those at risk of falls and those requiring assistance with ADL.

### 6 Minute Walk Test

The 6 minute walk is a measure of functional capacity that includes, but is not limited to, endurance, fatigability and cardiovascular fitness[38]. Our results emphasize that functional exercise capacity for pwHAM is severely impaired compared to predicted age matched normative values[20]. The increase in median distance walked between the 2 time points, was not expected in pwHAM., None of the patients were actively engaged in a physical activity or rehabilitation programme. Only 1 patient was on disease modifying therapy at both timepoints and 3 patients had initiated disease modifying therapy at the second time point.

However the increased distance could be attributed to the 6 patients who had a dramatic increase in their distances walked, of which 2 were on disease modifying therapies at the second time point. When these 6 patients are removed the median distance walked increased by only 6metres. Although age, weight, height and gender might be expected to contribute to the variability in the distance walked in 6 minutes in healthy adults[20], this was not the case here.

In pwHAM, as in pwMS and pwStroke, the distance covered in the 6MW and the time taken to walk 10m, are highly, inversely correlated[39].This potentially implies that the same aspect of walking capacity is being measured[38;39;40]. However using the 10m velocity to predict the 6 minute distance significantly over estimated the latter in our cohort. This might be due to the inability of pwHAM to maintain their walking velocity. The 10mTW and the 6MWT appear to represent different indicators of walking efficacy in pwHAM, with the longer 6MWT potentially capturing fatigue that is not picked up by the shorter measure. Therefore using the 10MTW and 6MWT concurrently, for our pwHAM, does not support the notion of redundancy.

### Other Factors

Walking speed is further influenced by the use of walking aids, as demonstrated by the strong correlation, at both time points, in our pwHAM. Our data agree with the studies reviewed by Hornyak et al[8] indicating that the use of a walking aid may modify the relationship between walking disability and self- selected walking speed[40], further masking the extent of their neuro-disability.

## Limitations to the Study

Space is at a premium in a clinical setting and in our clinic, we only have access to a 13.5m long smooth corridor. Shorter corridor lengths necessitate more turns and this may result in additional energy loss, which may have been the case in our patient sample[18;41]. As with most rare diseases, patient numbers for research purposes may be small, and the small sample size of our patient cohort attests to this, therefore lacking sufficient numbers to adequately power the study. Although the data points were a median of 18 months apart, patients were seen more frequently (monthly to 3 monthly) and the walking tests were conducted at these visits, potentially contributing to a learning effect.

## Summary

The functional walking capacity of pwHAM can be usefully measured using the 10mTW for gait speed and the 6MWT for endurance. Due to their functional deterioration, pwHAM mainly transition from community ambulators to household ambulators. The severity of their neuro-disability is highlighted by their slower household gait speeds, high dependence for ADL’s, a 1.5 fold greater likelihood of falling and insufficient gait speed to cross a road. Importantly, using 10m gait speed to predict the 6MW distance, overestimates the actual distance pwHAM walked. PwHAM have lower functional walking capacity compared to pwMS and age, weight, height and gender does not explain the variability in the 6MWT. The median deterioration in gait speed over 18 months is double the recommended minimal detectable change score of 0.1m/second.

## Conclusion

10mTW and 6MWT in pwHAM provide distinct information and significant changes can be detected over a short a period as 11 months and therefore these assessments can be useful to detect improvement or deterioration within broader disability grades. The 10mTW velocity underestimated the degree of disability. Comparison with published healthy age matched controls show that these patients have significantly slower gait speeds. Gait speed predicted functional ambulatory capacity and risk related to falls and dependence on ADL and shows direction of functional change. Walking capacity in pwHAM Walking capacity in pwHAM should be measured using the 10mTW for gait speed and the 6MW for endurance. Therefore the routine use of these measures in pwHAM could be used to formulate baseline assessments, allow comparisons over time, as well be as used in prospective clinical trials.

## References

[1] Gessain A, Cassar O. Epidemiological aspects and world distribution of HTLV-1 infection. Frontiers in Microbiology, 2012, 15;3: 388 10.3389/fmicb.2012.00388 23162541 PMC3498738

[2] De Castro-Costa CMD, Araújo AQ, Barreto MM, Takayanagui OM, Sohler MP, Silva E L D et al Proposal for diagnostic criteria of tropical spastic paraparesis/HTLV-I-associated myelopathy (TSP/HAM). AIDS Research & Human Retroviruses, 2006, 22;10: 931–935. 17067261 10.1089/aid.2006.22.931

[3] Report of the Scientific Group on HTLV-1 Infections and Associated Diseases. WHO Report (WP) CDS (S)/ICP/PHC/014.1988.

[4] Franzoi AC, Araújo AQC Disability profile of patients with HTLV-I-associated myelopathy/tropical spastic paraparesis using the functional independence measure (FIM™). Spinal Cord, 2005 43;4: 236–240. 15520834 10.1038/sj.sc.3101677

[5] Franzoi AC, Araújo AQC Disability and determinants of gait performance in tropical spastic paraparesis/HTLV-I associated myelopathy (HAM/TSP). Spinal Cord, 2007,45;1: 64–68. 16568145 10.1038/sj.sc.3101919

[6] Shublaq M, Orsini M, Puccioni-Sohler M. Implications of HAM/TSP functional incapacity in the quality of life. Arquivos de neuro-psiquiatria,2011, 69(2A): 208–211. 21537562 10.1590/s0004-282x2011000200013

[7] Schmid AA, Van Puymbroeck M, Altenburger PA, Dierks TA, Miller KK, Damush TM et al Balance and balance self-efficacy are associated with activity and participation after stroke: a cross-sectional study in people with chronic stroke. Archives of Physical Medicine and Rehabilitation, 2012, 93;6: 1101–1107. 10.1016/j.apmr.2012.01.020 22502804

[8] Hornyak V, VanSwearingen JM, Brach JS Measurement of gait speed. Topics in Geriatric Rehabilitation, 2012, 28;1: 27–32.

[9] Studenski S. Bradypedia: is gait speed ready for clinical use?. The Journal of Nutrition, Health & Aging, 2009, 13;10: 878–880. 10.1007/s12603-009-0245-0 PMC12878062 19924347

[10] Salbach NM, Mayo NE, Wood-Dauphinee S, Hanley JA, Richards CL, Cote R. A task-orientated intervention enhances walking distance and speed in the first year post stroke: a randomized controlled trial. Clinical Rehabilitation,2004, 18;5: 509–519. 15293485 10.1191/0269215504cr763oa

[11] Kempen JCE, de Groot V, Knol DL, Polman CH, Lankhorst GJ, Beckerman H. Community walking can be assessed using a 10-metre timed walk test. Multiple Sclerosis Journal, 2011, 17;8: 980–990. 10.1177/1352458511403641 21622593

[12] Amatachaya S, Naewla S, Srisim K, Arrayawichanon P, Siritaratiwat W. Concurrent validity of the 10-meter walk test as compared with the 6-minute walk test in patients with spinal cord injury at various levels of ability. Spinal Cord, 2014, 52;4: 333–336. 10.1038/sc.2013.171 24445972

[13] Graham JE, Ostir GV, Fisher SR, Ottenbacher KJ. Assessing walking speed in clinical research: a systematic review. Journal of Evaluation in Clinical Practice, 2008 14;4: 552–562. 10.1111/j.1365-2753.2007.00917.x 18462283 PMC2628962

[14] Wade DT, Wood VA, Heller A, Maggs J, Langton HR. Walking after stroke. Measurement and recovery over the first 3 months. Scandinavian Journal of Rehabilitation Medicine, 1986 19;1: 25–30. 3576138

[15] Martin F, Fedina A, Youshya S, Taylor GP A 15-year prospective longitudinal study of disease progression in patients with HTLV-1 associated myelopathy in the UK. Journal of Neurology, Neurosurgery & Psychiatry, 2010,81;12:1336–40 10.1136/jnnp.2009.191239 20660921

[16] Dourado VZ. Reference equations for the 6-minute walk test in healthy individuals. Arquivos brasileiros de cardiologia, 2011 96;6: e128–e138. 21359481

[17] Solway S, Brooks D, Lacasse Y, Thomas S. A qualitative systematic overview of the measurement properties of functional walk tests used in the cardiorespiratory domain. Chest Journal,2001, 119;1: 256–270. 10.1378/chest.119.1.256 11157613

[18] American Thoracic Society (ATS): Guideline for the Six minute walk test, American Journal of Respiratory and Critical Care Medicine, 2002,166;1:111–117 12091180 10.1164/ajrccm.166.1.at1102

[19] Guyatt GH, O Pugsley S, Sullivan MJ, Thompson PJ, Berman L, Jones NL et al Effect of encouragement on walking test performance. Thorax 1984,39;11:818–822. 6505988 10.1136/thx.39.11.818 PMC459930

[20] Troosters T, Gosselink R, Decramer M. Six minute walking distance in healthy elderly subjects. European Respiratory Journal, 1999, 14;2: 270–274. 10515400 10.1034/j.1399-3003.1999.14b06.x

[21] Bohannon RW, Andrews AW. Normal walking speed: a descriptive meta-analysis. Physiotherapy,2011, 97;3:182–189. 10.1016/j.physio.2010.12.004 21820535

[22] Perry J, Garrett M, Gronley JK, Mulroy SJ. Classification of walking handicap in the stroke population. Stroke, 1995, 26;6: 982–989 7762050 10.1161/01.str.26.6.982

[23] Fritz S, Lusardi M. White paper:“walking speed: the sixth vital sign”. Journal of Geriatric Physical Therapy, 2009, 32;2:2–5. 20039582

[24] Asher L, Aresu M, Falaschetti E, Mindell J. Most older pedestrians are unable to cross the road in time: a cross-sectional study. Age and Ageing,2012, 41;5: 690–694. 10.1093/ageing/afs076 22695790

[25] Schmid A, Duncan PW, Studenski S, Lai SM, Richards L, Perera S, et al Improvements in speed-based gait classifications are meaningful. Stroke, 2007, 38;7: 2096–2100. 17510461 10.1161/STROKEAHA.106.475921

[26] Studenski S, Perera S, Patel K, Rosano C, Faulkner K, Inzitari M et al "Gait Speed and Survival in Older Adults." JAMA 2011, 305;1: 50–58. 10.1001/jama.2010.1923 21205966 PMC3080184

[27] Peel NM, Kuys SS, Klein K. Gait speed as a measure in geriatric assessment in clinical settings: a systematic review. The Journals of Gerontology Series A: Biological Sciences and Medical Sciences, 2012,68;1: 39–46. 10.1093/gerona/gls174 22923430

[28] Abellan Van Kan G, Rolland Y, Andrieu S, Bauer J, Beauchet O, Bonnefoy Ml. Gait speed at usual pace as a predictor of adverse outcomes in community-dwelling older people: An International Academy on Nutrition and Aging (IANA) Task Force. Journal of Nutrition, Health and Aging. 2009;13:881–889. 19924348 10.1007/s12603-009-0246-z PMC12878092

[29] Studenski S, Perera S, Wallace D, Chandler JM, Duncan PW, Rooney E et al Physical performance measures in the clinical setting. Journal of the American Geriatrics Society, 2003,51;3:314–322. 12588574 10.1046/j.1532-5415.2003.51104.x

[30] Kuo HK, Leveille SG, Yen CJ, Chai HM, Chang CH, Yeh YC, et al Exploring how peak leg power and usual gait speed are linked to late-life disability: data from the National Health and Nutrition Examination Survey (NHANES), 1999–2002. American Journal of Physical Medicine & Rehabilitation,2006, 85;8:650–658 16865019 10.1097/01.phm.0000228527.34158.ed PMC2366087

[31] Bohannon RW, Andrews AW. Normal walking speed: a descriptive meta-analysis. Physiotherapy,2011, 97;3:182–189. 10.1016/j.physio.2010.12.004 21820535

[32] Bohannon RW. Comfortable and maximum walking speed of adults aged 20–79 years: reference values and determinants. Age and Ageing, 1997,26; 1:15–19. 9143432 10.1093/ageing/26.1.15

[33] Bohannon RW. Population representative gait speed and its determinants. Journal of Geriatric Physical Therapy, 2008, 31;2:49–52. 19856549 10.1519/00139143-200831020-00002

[34] Weiss RJ, Wretenberg P, Stark A, Palmblad K, Larsson P, Gröndal L. et al Gait pattern in rheumatoid arthritis. Gait & Posture, 2008,28;2:229–234. 18226528 10.1016/j.gaitpost.2007.12.001

[35] Middleton A, Fritz SL, Lusardi M. Walking speed: the functional vital sign. Journal of Aging and Physical Activity, 2015,23;2: 314–322. 10.1123/japa.2013-0236 24812254 PMC4254896

[36] Verghese J, Holtzer R, Lipton RB, Wang C. Quantitative gait markers and incident fall risk in older adults. The Journals of Gerontology Series A: Biological Sciences and Medical Sciences,2009, 64;8:896–901. 10.1093/gerona/glp033 PMC2709543 19349593

[37] Facchinetti LD, Araújo AQ, Chequer GL, de Azevedo MF, de Oliveira RVC, Lima MA. Falls in patients with HTLV-I-associated myelopathy/tropical spastic paraparesis (HAM/TSP). Spinal Cord, 2013, 51;3: 222–225. 10.1038/sc.2012.134 23165507

[38] Dobkin BH. Short-distance walking speed and timed walking distance: redundant measures for clinical trials?. Neurology, 2006,66;4:584–586. 16505318 10.1212/01.wnl.0000198502.88147.dd

[39] Dalgas U, Severinsen K, Overgaard K. Relations between 6 minute walking distance and 10 meter walking speed in patients with multiple sclerosis and stroke. Archives of Physical Medicine and Rehabilitation, 2012, 93;7:1167–1172. 10.1016/j.apmr.2012.02.026 22421626

[40] Dean CM, Richards CL, Malouin F. Task-related circuit training improves performance of locomotor tasks in chronic stroke: a randomized, controlled pilot trial. Archives of Physical Medicine and Rehabilitation, 2000 81;4: 409–417. 10768528 10.1053/mr.2000.3839

[41] Gibbons WJ, Fruchter N, Sloan S, Levy RD. Reference values for a multiple repetition 6-minute walk test in healthy adults older than 20 years. Journal of Cardiopulmonary Rehabilitation and Prevention, 2001,21;2: 87–93. 10.1097/00008483-200103000-00005 11314289

